# Transdermal patches loaded with etoricoxib nanocrystals as a remedy and prophylactic approach against progression of knee osteoarthritis

**DOI:** 10.1007/s13346-026-02052-6

**Published:** 2026-02-25

**Authors:** Lamiaa M. Ahmed, Omar H. Hosny, Zainab S. Abdelqader, Fergany A. Mohamed, Tahani H. Elfaham

**Affiliations:** 1 Department of Pharmaceutics, Faculty of Pharmacy, Assiut University, Assiut, 71526 Egypt; 2 Department of Surgery, Anesthesiology and Radiology, Faculty of Veterinary Medicine, Assiut University, Assiut, 71526 Egypt; 3 Department of Histology and Cell Biology, Faculty of Medicine, Assiut University, Assiut, 71526 Egypt

**Keywords:** Osteoarthritis, Etoricoxib nanocrystals, Transdermal, In vivo

## Abstract

Osteoarthritis (OA) is a heterogeneous and multifactorial disorder that affects the entire joint organ. It is a major global public health concern, impacting more than 500 million individuals worldwide. The onset and progression of OA are driven by a complex interplay of factors and modifiable risks such as obesity and joint injury. Consequently, OA imposes a substantial burden on patients’ quality of life and on society, owing to increased healthcare expenditures and reduced work productivity. The purpose of this study is to assess the therapeutic efficacy of transdermal patches loaded with etoricoxib nanocrystals (ETX-NCs, previously prepared and evaluated) in the treatment and reduction of osteoarthritis exacerbation. ETX-NCs patches of various polymers were prepared using solvent evaporation technique. The prepared patches were evaluated for drug content, thickness, moisture uptake, folding endurance, in vitro drug release, and skin permeation properties. The prepared patches based on HPMC, CMC Na, and PVA demonstrated uniformity, flexibility, smooth surface morphology and high drug content, along with acceptable physicochemical properties. Among these, the CMC Na based nanocrystal patches exhibited the most prolonged drug release (73.76 ± 2.38%). HPMC and CMC Na based patches showed promising skin penetration of 79.64 ± 1.20 μg/cm^2^ and 44.06 ± 2.72 μg/cm^2^, with corresponding flux values of 16.13 ± 0.21 μg/cm^2^/h and 8.08 ± 0.47 μg/cm^2^/h, respectively. Based on in vivo findings, the prepared ETX-NCs patches found to successfully alleviate OA symptoms within short duration (5 days), also protecting against disease progression.

**Graphical abstract:**

**Figure:**
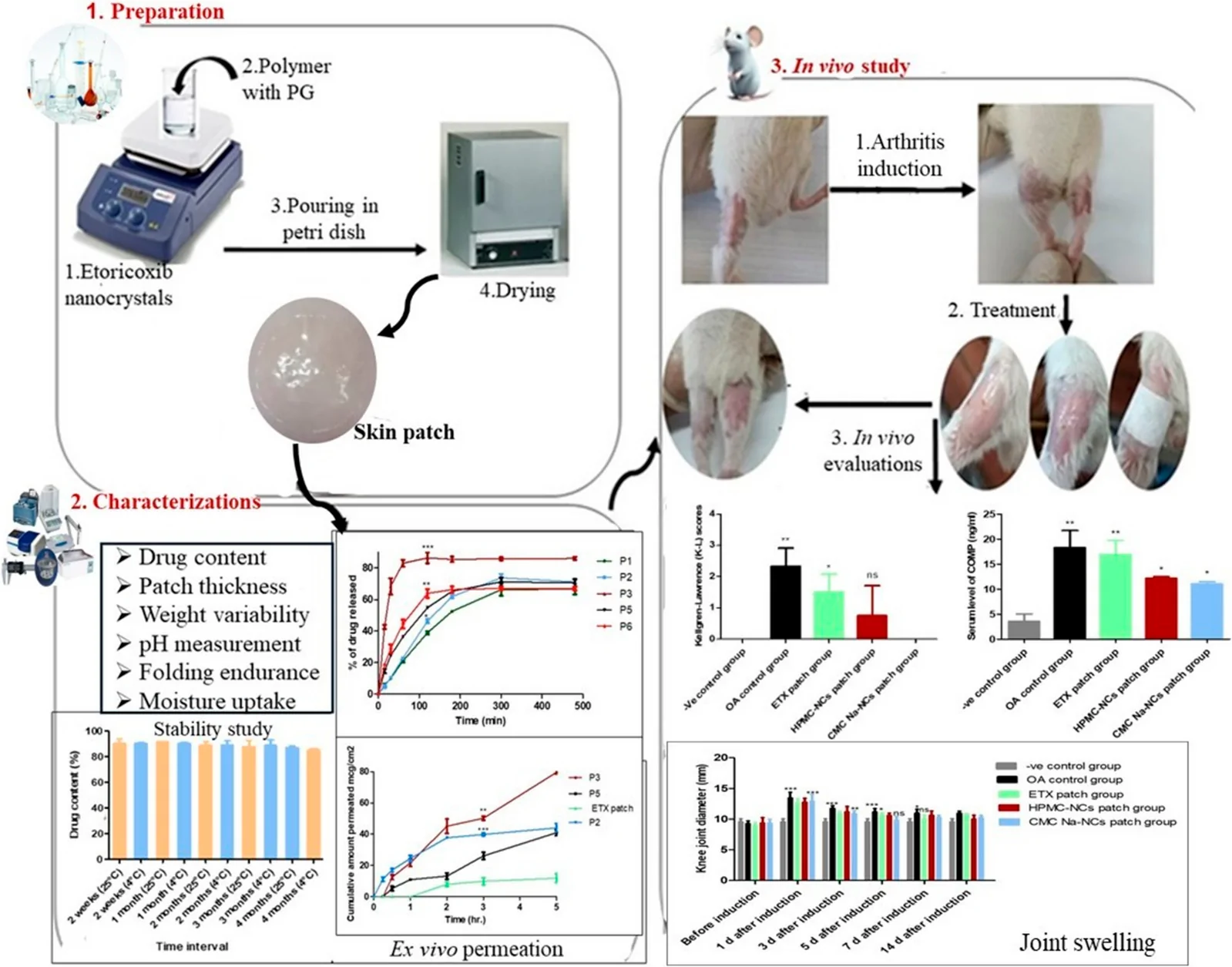

## Introduction

Osteoarthritis (OA) is the most prevalent chronic joint disorder worldwide, characterized by joint pain, inflammation, and progressive degeneration of cartilage, subchondral bone, menisci, and ligaments. Although the precise etiology of OA remains unclear, growing evidence underscores the pivotal role of inflammation in its onset and progression [1, 2]. Patients with OA often experience limited mobility, which severely restricts their participation in daily activities and are associated with reduced quality of life and psychological burden [3]. Approximately 73% of people with OA are over the age of 55, according to epidemiological data, and the prevalence is higher in women (about 60%). In developing nations, it is noteworthy that younger populations are increasingly experiencing a rising incidence of OA [4]. Projections from 2020 to 2050 predict a substantial global rise in OA prevalence, driven by population aging, demographic growth, and changing disease patterns, with the greatest increase anticipated in sub-Saharan Africa [4, 5]. According to the World Health Organization (WHO), OA management involves a multimodal approach including lifestyle modification, physical therapy, pharmacological treatment, and surgery based on disease severity. However, current therapies are limited by safety concerns, suboptimal efficacy, and poor patient adherence [5–7]. Consequently, there is a pressing need to develop novel pharmaceutical formulations that enhance patient compliance while minimizing adverse effects associated with conventional OA therapies. Etoricoxib (ETX) is a non-steroidal anti-inflammatory drug (NSAID) characterized by high selectivity for cyclooxygenase-2 (COX-2) inhibition, providing potent anti-inflammatory and analgesic effects with a lower incidence of adverse effects compared to COX-1 inhibitors [8, 9]. It was selected for this study due to its superior efficacy in managing osteoarthritis and arthritis at comparatively lower doses than other NSAIDs [10–12]. Nonetheless, its clinical use is limited by poor aqueous solubility, slow dissolution, and cardiovascular toxicity risks associated with oral administration [8, 13]. These limitations were successfully addressed through ETX formulation as nanocrystals (ETX-NCs), which significantly improved its solubility and dissolution rates as described in our previous work [14]. Transdermal delivery of ETX-NCs represents a promising route that may reduce systemic toxicity while improving therapeutic outcomes. Transdermal patches for drug delivery have garnered significant interest over the past decade owing to their numerous therapeutic advantages [15–17]. Transdermal administration as a noninvasive route offers an effective and patient-friendly alternative to conventional drug delivery routes [18]. Natural polymers like alginate, cellulose, gelatin, and pectin are highly suitable for patches preparation due to their excellent biocompatibility and capacity to form durable and flexible adhesion with the skin. Their natural origin helps to reduce the likelihood of skin irritation and unwanted side effects [15]. This present study evaluates the potential of using transdermal patches containing etoricoxib nanocrystals (ETX-NCs) for the effective management of osteoarthritis.

## Materials and methods

### Materials

Etoricoxib (EX) was a kind gift from APEX PHARMA, Cairo, Egypt, Hydroxy Propyl Methyl Cellulose E15 (HPMC E15) was obtained from alpha Chem Co., Cairo, Egypt. Sodium Carboxy Methyl Cellulose (CMC Na) was acquired from El Nasr Pharmaceutical Chemicals Co. Egypt, Sodium alginate was obtained from Alpha Chemika, Cairo, Egypt. PVA was procured from AVI CHEM LABORATORIES, Egypt, Monosodium iodo-acetate (MIA) was purchased from sigma-Aldrish. St. Louis, MO, USA. All other chemicals were of analytical grade and utilized as received.

### Methods

#### Preparation of transdermal patches of ETX-NCs

Based on findings from our previous work of preparing and evaluating etoricoxib nanocrystals (ETX-NCs), the optimized formula (30 mg of etoricoxib stabilized with 0.1% w/v lecithin at pH 5.5 and homogenized at 21,000 rpm for 5 min) exhibited a mean particle size of 210.3 ± 10.2 nm, a polydispersity index of 0.277 ± 0.005, and a zeta potential of − 74.1 ± 0.61 mV with a distinct cubic crystalline structure [14]. It was subsequently incorporated into different transdermal patch formulations. Patches of various polymers (HPMC E15, CMC Na, PVA, and sodium alginate, at two different amounts 150 mg and 250 mg for each polymer) were prepared and assessed, Table 1. Patches compositions were selected based on outcomes from preliminary experimental trials for patch preparation. All formulations were prepared using the solvent evaporation technique [19]. A defined weight of each polymer was incorporated into 11 mL of the nanocrystal (NCs) dispersion under continuous magnetic stirring for 2 h. Subsequently, 1% of propylene glycol (PG) was added as a plasticizer and permeation enhancer and stirring was continued for an additional one hour. The resulting mixtures were then poured into petri dishes (surface area: 50.24 cm^2^) and were allowed to dry in an oven at 40 °C overnight. The prepared patches were cut into smaller pieces (1 cm^2^) and were wrapped in aluminum foil till subsequent evaluations. In case of patches prepared with PVA, the polymer solution was stirred overnight until a clear solution was obtained, then poured and dried under the same conditions.

**Table 1:** Composition of ETX-NCs patches

Formula no	CMC Na. (mg)	HPMC E15 (mg)	PVA (mg)	Na alginate (mg)	PG (% v/v)
P1	150	-	-	-	1%
P2	250	-	-	-	1%
P3	-	150	-	-	1%
P4	-	250	-	-	1%
P5	-	-	150	-	1%
P6	-	-	250	-	1%
P7	-	-	-	250	1%
P8	-	250	-	250	1%

#### Characterization of the prepared ETX-NCs patches

##### Physicochemical characteristics

The prepared patches were visually inspected for uniformity, transparency, surface smoothness, and overall clarity.

**Patch thickness** was measured at four distinct points using a digital caliper, readings were recorded in millimeters (up to 10 mm scale) [20].

**Weight variability**; to assess weight uniformity, three film samples (1 cm^2^ each) were excised from different areas of the patch and were weighed using digital balance to verify consistency in patch composition [21].

**Folding endurance** was evaluated by repeatedly folding each 1 cm^2^ patch at the same location until it showed signs of breakage. The number of folds sustained without cracking was recorded, serving as an indicator of the patch’s flexibility and mechanical strength [22].

**Moisture uptake (%)** was determined by initially weighing each freshly prepared 1 cm^2^ patch, followed by storage at room temperature (25°C) for 3 days under controlled humidity conditions using a saturated potassium chloride solution chamber (84% relative humidity). The patches were reweighed at specific time intervals of 24, 48, and 72 h. The percentage of moisture absorbed was calculated using the following equation [23]:

1 $$Moisture\;uptake\;\%=\left(final\;weight-intial\;weight\right)/intial\;weight\times100$$

**pH measurement** was performed by dissolving two prepared patches (each measuring 1 cm^2^) in 3 ml of distilled water under magnetic stirring. The resulting solution was then measured using pH meter [24].

**Drug content**; three smaller patches pieces from different areas of the whole patch, each one of 1 cm^2^, were dissolved separately in 25 ml of buffer-methanol mixture (pH 7.4) at a ratio of 9:1. Initially, 2.5 ml of methanol were added to the patch and vortexed for 4 min, followed by the gradual addition of 22.5 ml of phosphate buffer. The resulting solutions were stirred using magnetic stirrer for 1 h to ensure complete dissolution. The absorbance was then measured using a UV spectrophotometer at 235 nm, and the drug content percentage was calculated using the following equation [24]:

2 $$Drug\;content\;\%=practical\;amount/theoretical\;amount\times100$$

**Differential Scanning Calorimetry (DSC) and Fourier Transform Infrared (FT-IR) spectroscopy** were employed to evaluate the physical state of etoricoxib and to assess possible interactions between ETX and formulation excipients within the prepared patches. DSC and FT-IR analyses were conducted on pure ETX, lecithin, HPMC, CMC Na, PVA, ETX-NC-loaded polymeric patches, and the corresponding physical mixtures of patch components (mixed at ratio of 1:1). For DSC measurements, approximately 5 mg of each sample was accurately weighed, sealed in aluminum pans, and heated at constant rate of 10°C/min over a temperature range of 25–300°C using a computer-controlled Shimadzu differential scanning calorimeter.

FT-IR spectra were recorded using an FT-IR spectrophotometer (Shimadzu IR-345, Japan). Samples were finely ground with potassium bromide, compressed into pellets, and scanned over the wavenumber range of 500–4000 cm⁻^1^ [25, 26].

##### In vitro drug release

The in vitro release rate of ETX from the prepared transdermal patches was assessed using the dialysis method. Patches with a surface area of 3.46 cm^2^ were placed in the donor compartment, which was configured using cellulose dialysis membrane (molecular weight cut-off (MWCO) 12,000–14,000 Da,flate width 44 mm Dry diameter 28 mm, Spectrum Labs Inc., USA). The receptor compartment contained 20 ml of phosphate buffer (pH 7.4), ensuring sink conditions. The assembly was maintained in a thermostatically controlled water bath at 37 ± 0.5°C with continuous agitation at 50 rpm. At predetermined time intervals, 2 ml aliquots were withdrawn from the receptor medium and immediately replaced with an equal volume of fresh buffer at the same temperature. The collected samples were analyzed spectrophotometrically at λmax of 235 nm. All experiments were performed in triplicate [27]. The in vitro drug release data were analyzed by fitting the results to various kinetic models to determine the release mechanism. These models included zero-order, first-order kinetics and the Higuchi model [28].

##### Ex vivo skin permeation study

The permeability of ETX through rat hairless skin was evaluated using patch samples with an area of 3.46 cm^2^. Male Wistar rats weighing between 200 ± 30 g were used, and the experimental protocol was conducted in accordance with the guidelines of the Ethical Committee of Faculty of Pharmacy, Assiut University (Approval No: 05–2023-022). Prior to the study, the rats were euthanized by cervical dislocation, and full-thickness dorsal skin was excised. The isolated skin was rinsed with distilled water followed by phosphate buffer (pH 7.4) [29]. Using two open-ended tubes, the skin was mounted on one end, such that the stratum corneum faced the donor compartment. The experiment was conducted in triplicate for each sample under conditions like those used in the in vitro release study. ETX permeation was quantified by UV spectrophotometric analysis at a wavelength of 235 nm, using blank samples prepared under identical conditions [30, 31]. The permeability coefficient (P_app_) was calculated by dividing the steady-state drug flux by the initial drug concentration in the formulation. The drug flux (Jss) was determined from the slope of the linear portion of the permeation curve, normalized to the exposed surface area of the skin [29].

##### Stability study

Stability study was conducted to reveal how storage affected the prepared patches. For four months, the transdermal prepared patches were incubated at room temperature (25 ± 2 °C) and at refrigeration temperature (4–8 °C). At regular intervals of two weeks, one month, two months, three months and four months, the prepared patches’ appearance, folding endurance and drug content were assessed [32].

#### In vivo study

##### Skin irritation test

A 48-h skin irritation study was conducted in twelve rats to assess the dermal safety of the selected patches. After hair removal from the dorsal skin 24 h prior to the experiment, rats were divided into four groups (n = 3): Group I served as the untreated control, while Groups II, III, and IV received 0.8% w/v formalin solution (positive control), CMC-Na NCs patch, and HPMC-NCs patch, respectively. Formalin was used as a positive control due to its well-known irritant properties [33]. Skin sites were evaluated for erythema and edema at 1, 6, 24, and 48 h post-application using the Draize scoring system (0–4, where 0 indicated no reaction, and scores of 1, 2, 3, and 4 represented very slight, slight, moderate, and severe reactions, respectively). Mean scores were calculated for each group, and the Primary Irritancy Index (PII) was determined by summing erythema and edema scores to compare dermal irritation among treatments [34–36].

##### Osteoarthritic model

Animals: Twenty-five male Wistar albino rats, weighing between 120 and 140 g, were obtained from faculty of Veterinary Medicine’s Animal Facility, Assiut University. The animals were kept in regular laboratory conditions, which included a 12-h light/12-h dark cycle and adequate ventilation, all rats had unrestricted access to food and water. The animals were given a week to accommodate their surroundings before the experiment began [37, 38]. All procedures involving animal handling, use, and euthanasia were approved by Research Ethics Committee of Faculty of Pharmacy, Assiut University (Approval No: 05–2023-022) which is a branch of Assiut University Research Ethics Committee (AUREC) and both conform with ARRIVE guidelines. To optimize osteoarthritis induction, preliminary experiments were carried out utilizing various rat weight ranges (180–220, 250–300 and 120-140g) and monosodium iodoacetate (MIA) dosages (2–4 mg/50 μL saline). joint histology, joint swelling evaluation, and serum inflammatory markers (TNF-α and IL-1β) all confirmed successful induction.

##### Experimental protocol

**Animal grouping:** The rats were randomly divided into five groups (n = 5 per group). Group 1 served as the negative control (healthy rats), representing the normal knee joint structure. Group 2 acted as the OA control group to evaluate the natural progression and self-recovery of osteoarthritis without any treatment. Group 3 received CMC Na-NCs patch formula (P2), Group 4 was treated with HPMC-NCs based patch formula (P3), and Group 5 administered the ETX patch (not in nanocrystal form). All treatments were applied from day two after MIA injection once daily for 14 days.

**Induction of osteoarthritis (OA):** Single unilateral intra-articular injection of monosodium iodoacetate (MIA) was used to induce osteoarthritis. All animals received an intraperitoneal injection of 5% ketamine solution (40–90 mg/kg) and 2% xylazine (5–10 mg/kg) to create general anesthesia [39]. The knee joint area was then shaved and disinfected, and a sterile 100-U insulin syringe was used to inject 3 mg of MIA dissolved in 50 μL of sterile saline (0.9%w/v) into the right knee joint cavity through the patellar ligament. Rats in the negative control group (healthy rats) received an equivalent volume (50 μL) of sterile saline instead of MIA [40, 41]. 

##### Evaluation of the anti-osteoarthritic effect

Determination of joint swelling: The swelling diameter of the right knee joint of each rat was measured using a caliper (mm scale) at multiple time intervals: before induction, and at 1, 3, 5, 7 and 14 days post-induction [42].

Serum analysis: The rats’ retro-orbital sinuses were used to withdraw blood samples 14 days after induction. After being allowed to clot for an hour at room temperature, the samples were centrifuged at 1000 × g for 20 min at 2–8°C. The resultant supernatant was gathered for examination. Key biomarkers of cartilage degradation, such as C-telopeptide of type II collagen (CTX-II) and cartilage oligomeric matrix protein (COMP), were measured in rat serum using commercially available enzyme-linked immunosorbent assay (ELISA) kits in accordance with the manufacturer’s instructions. Both were acquired from Elabscience^®^ Wuhan, China [43].

Radiographical examination: A radiographic evaluation was conducted on day 14. The rats were put under ketamine/xylazine-induced anesthesia and had X-ray imaging of their right stifle joint in the lateral (L) view and of both hind limbs in the anteroposterior (AP) view. Using a 50-mA fixed computed radiography (CR) X-ray machine, radiographs were obtained. All radiographic images were independently assessed in a blinded manner by two investigators. The Kellgren-Lawrence (K-L) grading method, which rates osteoarthritic alterations on a scale of 0 to 4, was used to grade the degree of joint degeneration where 0 = none, 1 = doubtful, 2 = mild, 3 = moderate, and 4 = severe [41, 44].

Histopathology: At the end of the experiment, all rats were sacrificed via neck dislocation after sedation using diethyl ether [45]. Joint samples were collected from the right knee joints of rats from each group and fixed in 10% neutral buffered formalin for 48 h. The samples were then decalcified in formic acid (10%) for 6 days [46, 47]. then joints were trimmed and were sectioned in the frontal (coronal) plane [48]. The tissues were subsequently processed through graded alcohol series for dehydration, cleared in xylene, and embedded in paraffin. Sections of 5 μm thickness were prepared using a rotary microtome and mounted on glass slides. These sections were stained with hematoxylin and eosin (H&E) for histological evaluation. Osteoarthritis Research Society International (OARSI) scoring system was used to assess the histopathological alterations across all groups. This system employs a six-grade scale to evaluate lesion severity and a four-stage scale to determine the extent of cartilage damage, with the overall score derived from the multiplication of grade and stage as reported by Pritzker et al. and Mostafa et al. [49, 50].

### Statistical analysis

GraphPad Prism software version 5 was used to develop statistics and graphical presentations. Statistical significance (* (P < 0.05), **(P < 0.01), ***(P < 0.001), and NS (non-significant) among groups was assessed using one-way analysis of variance (ANOVA) followed by the Newman–Keuls post hoc test. All experiments were performed in triplicate, except for anti-osteoarthritic evaluations, where n = 5. All data are presented as the mean ± standard deviation (SD). The Shapiro–Wilk test was used to assess data normality. No significant deviations were detected; however, the small sample size restricted the confidence of this assessment so graphical inspection of data distribution and homogeneity of the variances were also performed.

## Results and discussion

### Physicochemical characteristics

**Fig. 1:**
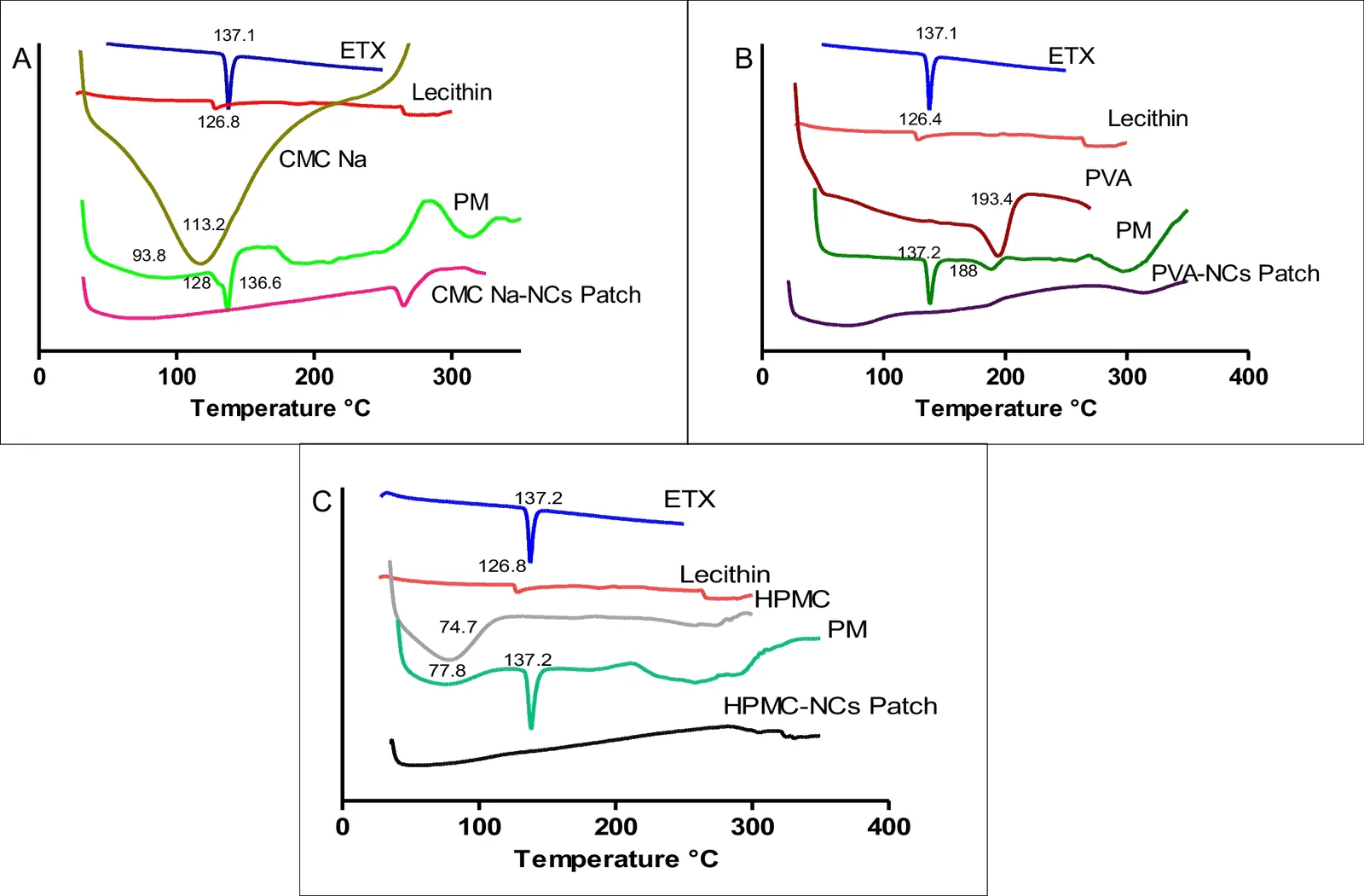
DSC thermograms of prepared patches, recorded at a rate of 10 °C/min over a temperature range of 25–300 °C, **A**) DSC thermogram of ETX, Lecithin, CMC Na, physical mixture (PM) and CMC Na-NCs patch, **B**) DSC thermogram of ETX, Lecithin, PVA, physical mixture (PM) and PVA-NCs patch, **C**) DSC thermogram of ETX, Lecithin, HPMC, physical mixture (PM) and HPMC-NCs patch

**Fig. 2:**
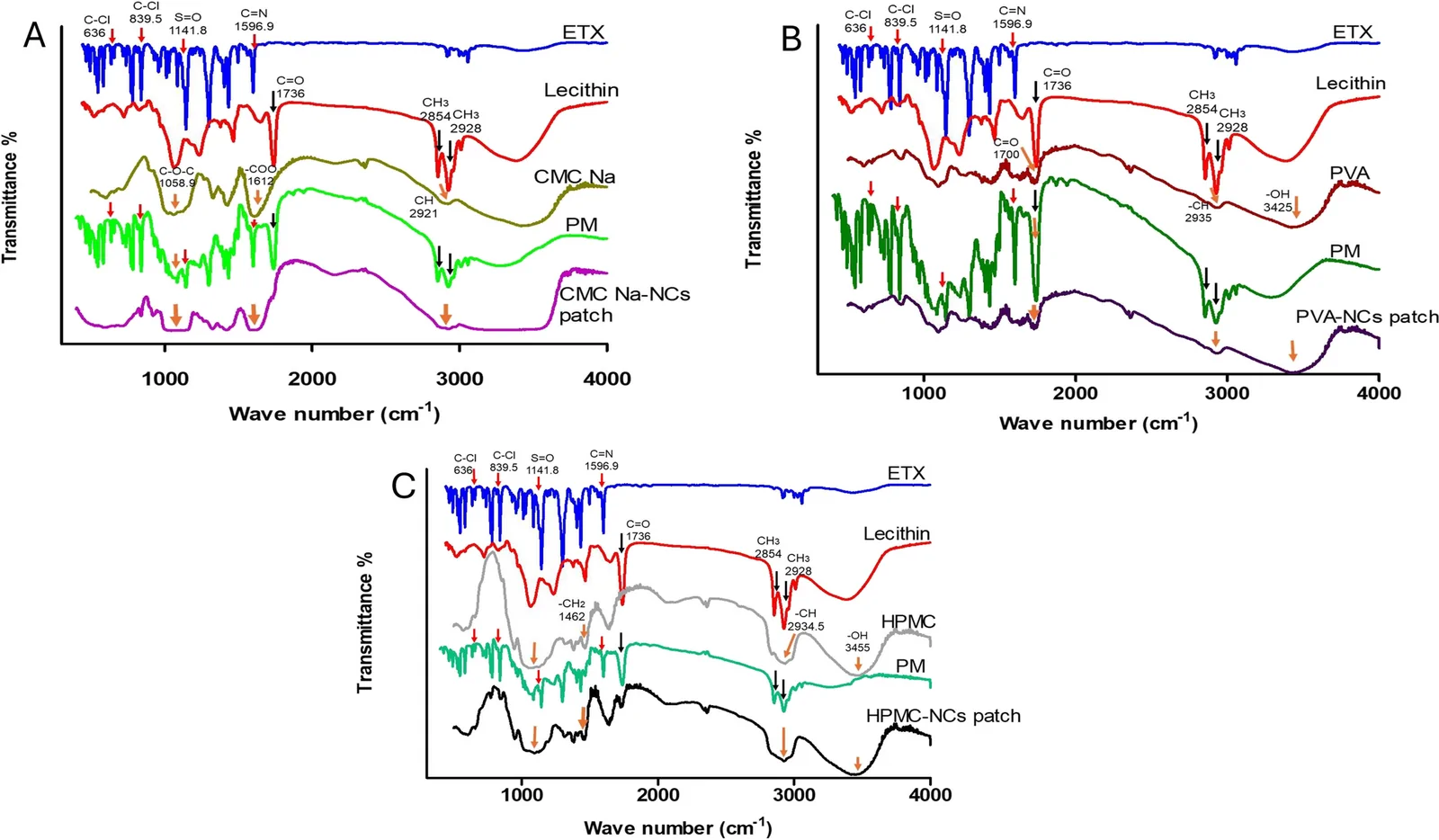
FT-IR spectra of prepared patches, **A**) FT-IR spectra of ETX, Lecithin, CMC Na, physical mixture (PM) and CMC Na-NCs patch, **B**) FT-IR spectra of ETX, Lecithin, PVA, physical mixture (PM) and PVA-NCs patch, **C**) FT-IR spectra of ETX, Lecithin, HPMC, physical mixture (PM) and HPMC-NCs patch

The prepared patches (P1-P6) were evaluated for their physical characteristics through visual inspection. All formulations appeared smooth, uniform and flexible with no signs of brittleness or phase separation. Patches formulated with sodium alginate (P7 and P8) exhibited excessive brittleness, consequently, they were excluded from further evaluations. The thickness of the prepared patches ranged from 0.09 ± 0.01 mm to 0.20 ± 0.02 mm, as shown in Table 2. An increase in polymer concentration accompanied with a proportional increase in patch thickness which was in accordance with Latif et al. [32]. Assessment of weight uniformity revealed consistent mass across formulations, with weights ranging from 4.60 ± 0.00 mg to 9.83 ± 0.00 mg (Table 2). All formulations exhibited high folding endurance (> 200 folds) without any cracking or breaking, consistent with the findings of Tadhi et al. [51]. Drug content analysis showed values ranging from 89.84 ± 2.71% to 104.41 ± 1.82%, with low standard deviation, indicating good content uniformity and the effectiveness of the solvent evaporation method in producing homogeneous films [30]. Transdermal patch P4 was excluded from further evaluations as it did not achieve content uniformity indicated with high SD value (93.16 ± 27.38). Moisture uptake across the formulations was low, ranging from 0.90 ± 0.76% to 6.55 ± 2.19%, which may contribute to reduced microbial susceptibility and prevention of patch bulkiness [52]. The surface pH of the patches was found to be within the neutral range (7.05 ± 0.07 to 7.72 ± 0.08), suggesting suitability for skin application without causing irritation (Table 2). To evaluate the compatibility of components used in the patch formulations, Differential Scanning Calorimetry (DSC) and Fourier Transform Infrared (FT-IR) analyses were conducted. The results of DSC showed distinct endothermic peaks at 137.1 °C for ETX and at 113.2 °C for sodium carboxymethyl cellulose (CMC-Na), Fig. 1A [53, 54]. These peaks were still present, albeit with a slight shift, in the thermogram of the physical mixture. However, drug characteristic peak disappeared completely in the thermogram of the formulated patch (CMC Na-NCs patch), Due to the small amount of ETX incorporated into the patch and suggesting successful drug encapsulation and molecular dispersion within the polymeric matrix [55]. Similar findings were noted for the other formulations. In the PVA-based nanocrystal patch (Fig. 1B), the endothermic peak associated with PVA at 193.4 °C was observed [56]. Likewise, the HPMC-based patch (Fig. 1C) showed the original HPMC peak at 74.7 °C [57]. FT-IR results are in harmony with the DSC findings as the characteristic fingerprint absorption bands of ETX (1596.9 cm⁻^1^ (C = N), 1141.8 cm⁻^1^ (S = O), 839.5 and 636 cm⁻^1^ (C–Cl) and lecithin (2854, 2928 cm⁻^1^ (CH₃) and 1736 cm⁻^1^ (C = O)) were retained in all physical mixtures spectra, Fig. 2 [58, 59]. In contrast, the spectra of ETX-NC-loaded patches were dominated by the characteristic bands of the polymers: CMC Na (C–O–C at 1058.9 cm⁻^1^ and –COO⁻ at 1612 cm⁻^1^), PVA (C–H at 2935 cm⁻^1^, –OH at 3425 cm⁻^1^, C = O at 1700 cm⁻^1^, (C–O)–C–OH at 1094.5 cm⁻^1^ and C–C at 849.7 cm⁻^1^) and HPMC (–CH₂ scissoring at 1462 cm⁻^1^, –CH stretching at 2934.5 cm⁻^1^, and –OH at 3455 cm⁻^1^), Fig. 2 [60–62].These results confirmed good compatibility between formulation components which conform with results obtained by Shehata et al. [63].

**Table 2:** Physicochemical characteristics of the prepared ETX-NCs patches

Patch	Polymer /amount	Physical appearance	Thickness (mm)	Weight variability (mg)	Folding endurance	Moisture uptake %	pH	Drug content %
P1	CMC Na 150 mg	Smooth, flexible, transparent	0.1 ± 0.00	5.26 ± 0.00	> 200	4.65 ± 1.13	7.72 ± 0.08	97.86 ± 5.35
P2	CMC Na 250 mg	Smooth, flexible, transparent	0.2 ± 0.05	8.06 ± 0.00	> 200	3.49 ± 0.84	7.63 ± 0.04	92.97 ± 1.57
P3	HPMC 150mg	Smooth, flexible, transparent	0.09 ± 0.01	4.60 ± 0.00	> 200	2.32 ± 0.07	7.56 ± 0.02	89.84 ± 2.71
P4	HPMC 250mg	Smooth, flexible, transparent	0.14 ± 0.02	9.83 ± 0.00	> 200	0.90 ± 0.76	7.53 ± 0.04	93.16 ± 27.38
P5	PVA 150mg	Smooth, flexible, transparent	0.12 ± 0.02	5.50 ± 0.00	> 200	6.55 ± 2.19	7.26 ± 0.05	104.41 ± 1.82
P6	PVA 250mg	Smooth, flexible, transparent	0.2 ± 0.02	7.16 ± 0.01	> 200	2.45 ± 0.45	7.05 ± 0.07	94.76 ± 9.28
P7	Na-alginate 250mg	Totally Fragile, ununiform	-	-	-	-	-	-
P8	HPMC + Na-alginate (1:1)	Totally Fragile, ununiform	-	-	-	-	-	-

### Drug release and release kinetics

**Fig. 3:**
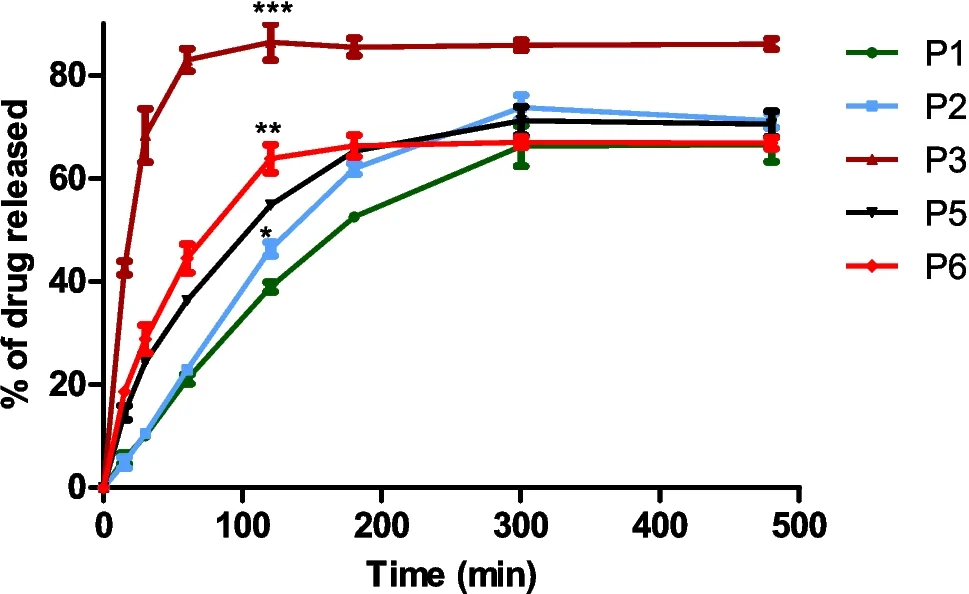
Release profiles of ETX from different prepared patches using natural hydrophilic polymers; P1(CMC Na 150 mg-NCs patch), P2 (CMC Na 250 mg-NCs patch), P3 (HPMC 150 mg-NCs patch), P5 (PVA 150 mg-NCs patch) and P6 (PVA 250 mg-NCs patch). The release medium was phosphate buffer (pH 7.4), Data are presented as mean ± SD (n = 3), Statistical analysis was performed using one-way ANOVA followed by Newman–Keuls post hoc test with significance indicated as *** (p < 0.001) between P3 and P2, ** (p < 0.01) between P6 and P5 and * (p < 0.05) between P2 and P1

Drug release from all transdermal patches was evaluated over 8 h using the dialysis method. The HPMC-based nanocrystal patch (P3) showed the fastest release, with 86.46 ± 3.51% released within 2 h (p < 0.001), significantly higher than P1 (38.93 ± 0.92%), P2 (46.34 ± 1.27%), P5 (54.90 ± 0.36%), and P6 (63.83 ± 2.73%) during the same period (Fig. 3). This rapid release may be attributed to the high hydrophilicity and swelling capacity of HPMC enhancing water uptake and drug diffusion which conform with the findings of Nandi and Mondal [64]. Furthermore, an overall trend of increased drug release with higher polymer concentration was observed (Fig. 3), which may be due to greater matrix hydration and polymer erosion at higher polymer levels, facilitating drug diffusion. These findings were consistent with Sampaopan et al. and Tirunagari et al. [65, 66]. In vitro release data were fitted to different kinetic models to elucidate the drug release mechanism. HPMC and PVA based formulations (P3, P5 and P6) showed the best fit to the Higuchi model, as evidenced by higher r^2^ values with n values below 0.5 (0.1426, 0.350, and 0.250, respectively, Table 3) indicating Fickian diffusion-controlled release. [64, 65, 67]. Similarly, CMC-Na-based formulations (P1,150 mg and P2,250 mg) followed the Higuchi model but n values of 0.626 and 0.622, indicating a non-Fickian (anomalous) release mechanism involving both diffusion and polymer relaxation or erosion. Varying the polymer concentration did not affect the release kinetics, although hydration and swelling were enhanced [32, 68].

**Table 3:** Release kinetic models of ETX from prepared patches

Patch	Zero order (r^2^)	First order (r^2^)	Second order (r^2^)	Diffusion model Higuchi (r^2^)	Korsmeyer-Peppas (r^2^)	Korsmeyer-Peppas (n)
P1	0.9015	0.9326	0.9497	**0.9664**	0.9616	**0.6259**
P2	0.8678	0.8997	0.9109	**0.9464**	0.9425	**0.6216**
P3	0.5610	−0.0891	0.6483	**0.76442**	0.7897	**0.1426**
P5	0.8367	0.8771	0.9063	**0.9298**	0.9466	**0.3504**
P6	0.7430	0.7692	0.7907	**0.8608**	0.8820	**0.2503**

### Ex vivo skin permeation

**Fig. 4:**
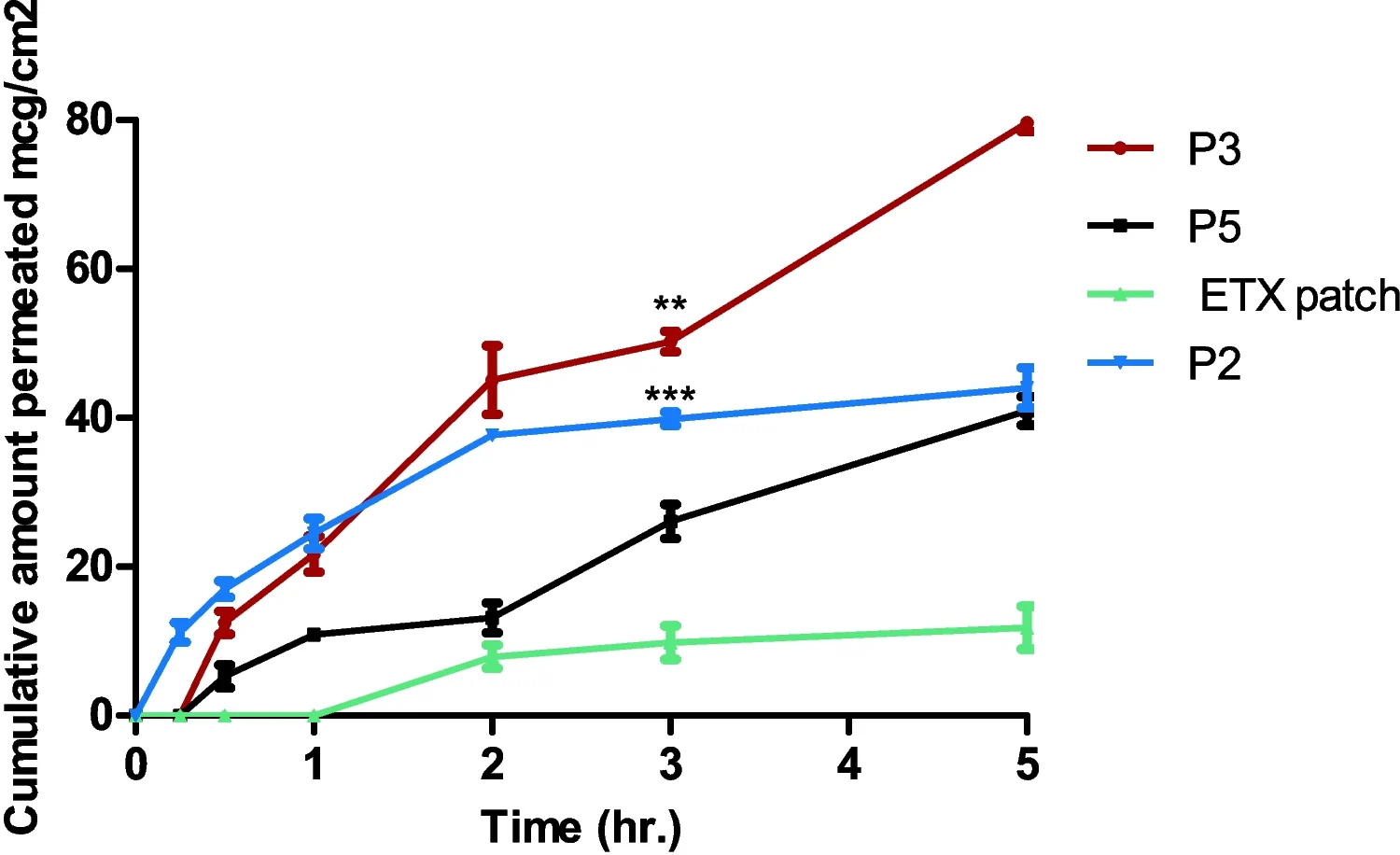
Ex vivo permeation profiles of ETX from different NCs patches against ETX patch, P2 (CMC Na 250 mg-NCs patch), P3 (HPMC 150 mg-NCs patch), P5 (PVA 150 mg-NCs patch), ** (p < 0.01) is the significance level between P3 and P2 while *** (p < 0.001) between P2 and ETX patch.Results are shown as mean ± SD (n = 3). One way ANOVA followed by the Newman–Keuls post hoc test was used for statistical analysis

The permeability of ETX via excised skin from various transdermal patches (P2,P3 and P5) was evaluated. The formulations were selected based on in vitro drug release performance. Figure 4 illustrates the cumulative drug permeation (µg/cm^2^) across rat skin over 5 h for patch formulations and the conventional ETX patch. Among all formulations, the HPMC-NCs patch (P3) showed the highest permeation, reaching 79.64 ± 1.20 µg/cm^2^ with a flux of 16.13 ± 0.21 µg/cm^2^/h, compared to P5 (40.94 ± 1.89 µg/cm^2^), the ETX patch (11.83 ± 2.86 µg/cm^2^), p < 0.001 and P2 (44.06 ± 2.72 µg/cm^2^, p < 0.01), Table 4. This may be attributed to hydrophilicity and high-water vapor permeability of HPMC, which enhances skin hydration and disrupts the stratum corneum lipid structure, thereby facilitating drug diffusion [69–71]. P2 (CMC Na-NCs patch) also demonstrated considerable skin permeation, second only to P3, highlighting its potential as another promising candidate formulation. The higher transdermal permeation observed with CMC Na compared to PVA, despite differences in their in vitro release profiles (these differences are not significant at all points, Fig. 3), may be attributed to the higher swelling capacity of CMC Na [72–74]. This enhanced property allows CMC Na-based patches to absorb more water, leading to greater expansion and formation of additional aqueous channels within the normal routes in the skin. Thus improved drug diffusion and increased transdermal permeation [75]. In contrast, the poor permeation of ETX patch (ETX not in nanocrystal form) underscores the advantage of nanocrystal formulations, which may increase drug saturation solubility and concentration gradient across the skin, thereby enhancing passive diffusion [76, 77]. Based on these results, P2 (CMC Na–NCs patch) and P3 (HPMC–NCs patch) were selected for in vivo evaluation to assess their therapeutic efficacy against knee OA.

**Table 4:** Ex vivo permeation parameters of ETX from different patch formulations through rat dorsal skin

Parameter	Q (mcg cm^−2^)	Jss (mcg cm^−2^ h^−1^)	P_app_x10 (cm/h)
Patch			
P2	44.06 ± 2.72	8.08 ± 0.47	0.59 ± 0.03
P3	79.64 ± 1.20	16.13 ± 0.21	1.17 ± 0.02
P5	40.94 ± 1.89	8.22 ± 0.52	0.60 ± 0.04
ETX-patch	11.83 ± 2.86	2.79 ± 0.66	0.32 ± 0.08

### Stability study

Stability study was performed on the CMC-Na NCs patch, selected for its sustained drug release and favorable skin permeation (Figs. 3 and 4). Patches were stored at room temperature (25 ± 2 °C) and under refrigeration (4–8 °C) for four months. No significant changes in drug content were observed over time or between different storage conditions (Table 5). The patches also maintained their physical integrity, remaining smooth, transparent and flexible with folding endurance > 200 throughout the study period. These findings demonstrate that the CMC-Na NCs patch is physically and chemically stable and can be safely stored at room temperature or under refrigeration for up to four months without significant alteration in its physicochemical properties.

**Table 5:** Storage effect on CMC Na-NCs patch at room temperature and refrigeration

Time interval	Room temperature (25 ± 2 °C)			Refrigeration (4–8 °C)		
	Appearance	Folding endurance	Drug content (%)	Appearance	Folding endurance	Drug content (%)
Zero time	Smooth, Transparent	> 200	89.51 ± 0.61	Smooth, Transparent	> 200	89.51 ± 0.61
2 weeks	Smooth, Transparent	> 200	90.15 ± 3.92	Smooth, Transparent	> 200	90.37 ± 0.61
1 month	Smooth, Transparent	> 200	91.44 ± 0.30	Smooth, Transparent	> 200	90.40 ± 0.42
2 months	Smooth, Transparent	> 200	88.66 ± 2.99	Smooth, Transparent	> 200	88.94 ± 3.20
3 months	Smooth, Transparent	> 200	87.80 ± 4.83	Smooth, Transparent	> 200	88.66 ± 4.23
4 months	Smooth, Transparent	> 200	86.95 ± 1.20	Smooth, Transparent	> 200	85.24 ± 1.21

### In vivo studies

#### Skin irritation test

**Fig. 5:**
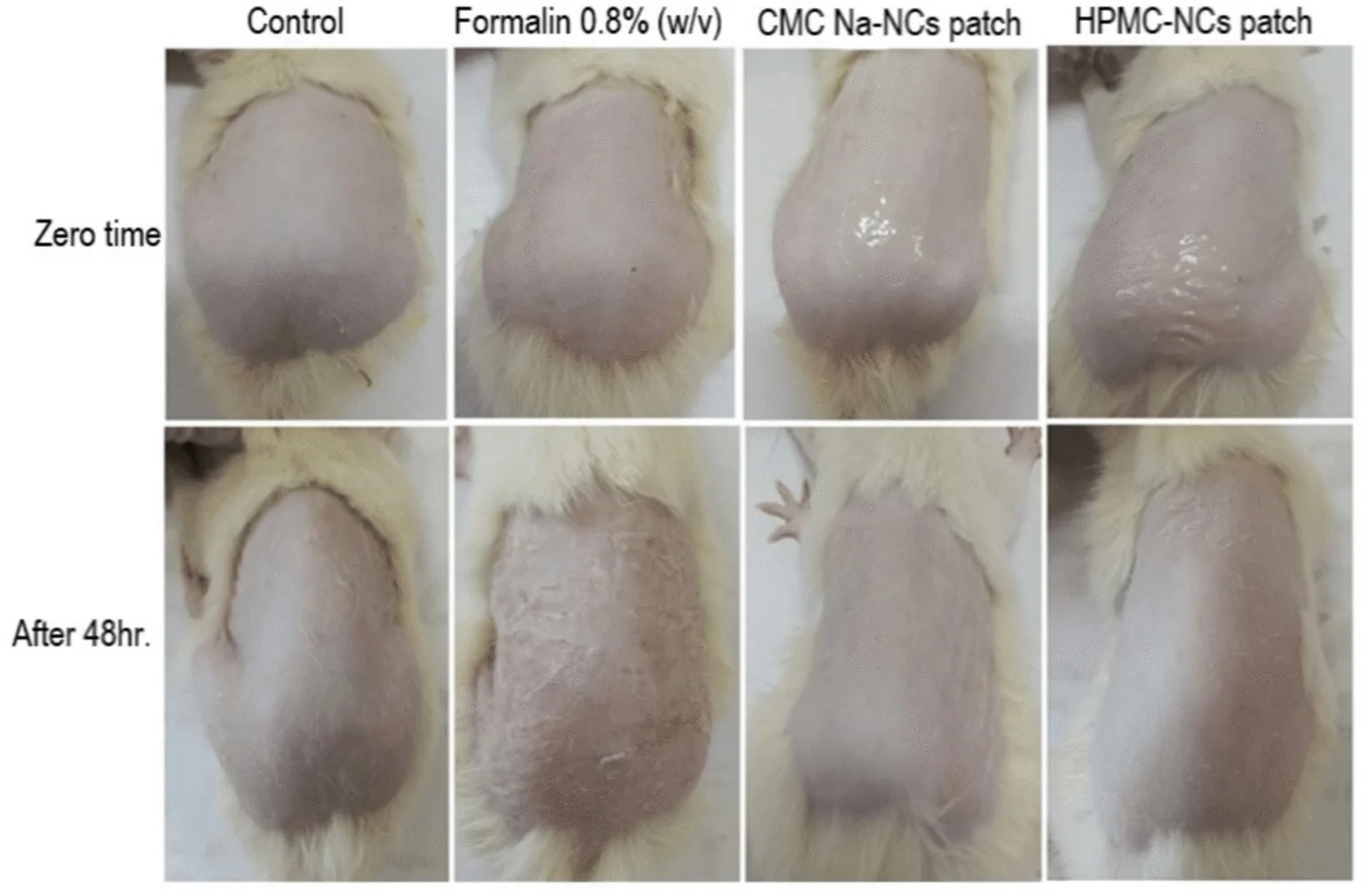
Representative photographs demonstrating skin responses (n = 3) following application of ETX-NCs patches (CMC Na-NCs and HPMC-NCs) in comparison with formalin (0.8% w/v) as a positive control, recorded at zero time and after 48 h

The irritation potential of the tested patches was evaluated, with the results summarized in Table 6 and illustrated in Fig. 5. The irritation evaluated by the mean erythema and edema scores, along with the calculated Primary Irritancy Index (PII) for each group. The group treated with 0.8% w/v formalin exhibited a PII of 4.83, indicating significant irritation. In contrast, groups III and IV, which received CMC-Na NCs patch and HPMC-NCs patch, showed markedly lower PII values of 0.16 and 0.08, respectively. According to these findings, the tested ETX-NCs (CMC-Na NCs and HPMC-NCs) patches are classified as non-irritant and safe for skin application, as their PII values remain below the threshold of 2, in accordance with the criteria established by Draize et al. [78, 79].

**Table 6:** Scores of skin irritation test

Time	Animals (*n* = 3)	Control		Formalin 0.8% (w/v)		CMC Na-NCs patch		HPMC-NCs patch	
		Erythema	Edema	Erythema	Edema	Erythema	Edema	Erythema	Edema
1 h	1	0	0	2	2	0	0	0	0
	2	0	0	2	1	0	0	0	0
	3	0	0	3	2	0	0	0	0
6 h	1	0	0	2	3	1	0	0	0
	2	0	0	2	1	0	0	0	0
	3	0	0	3	2	0	0	0	0
24 h	1	0	0	4	4	1	0	0	0
	2	0	0	3	1	0	0	0	0
	3	0	0	4	4	0	0	0	0
48 h	1	0	0	3	1	0	0	0	0
	2	0	0	3	1	0	0	1	0
	3	0	0	4	1	0	0	0	0
Mean score ± SD		0	0	2.91 ± 0.79	1.91 ± 1.16	0.16 ± 0.38	0.00 ± 0.00	0.08 ± 0.28	0.00 ± 0.00
P.I.I	0			4.83		0.16		0.08

#### Determination of joint swelling

**Fig. 6:**
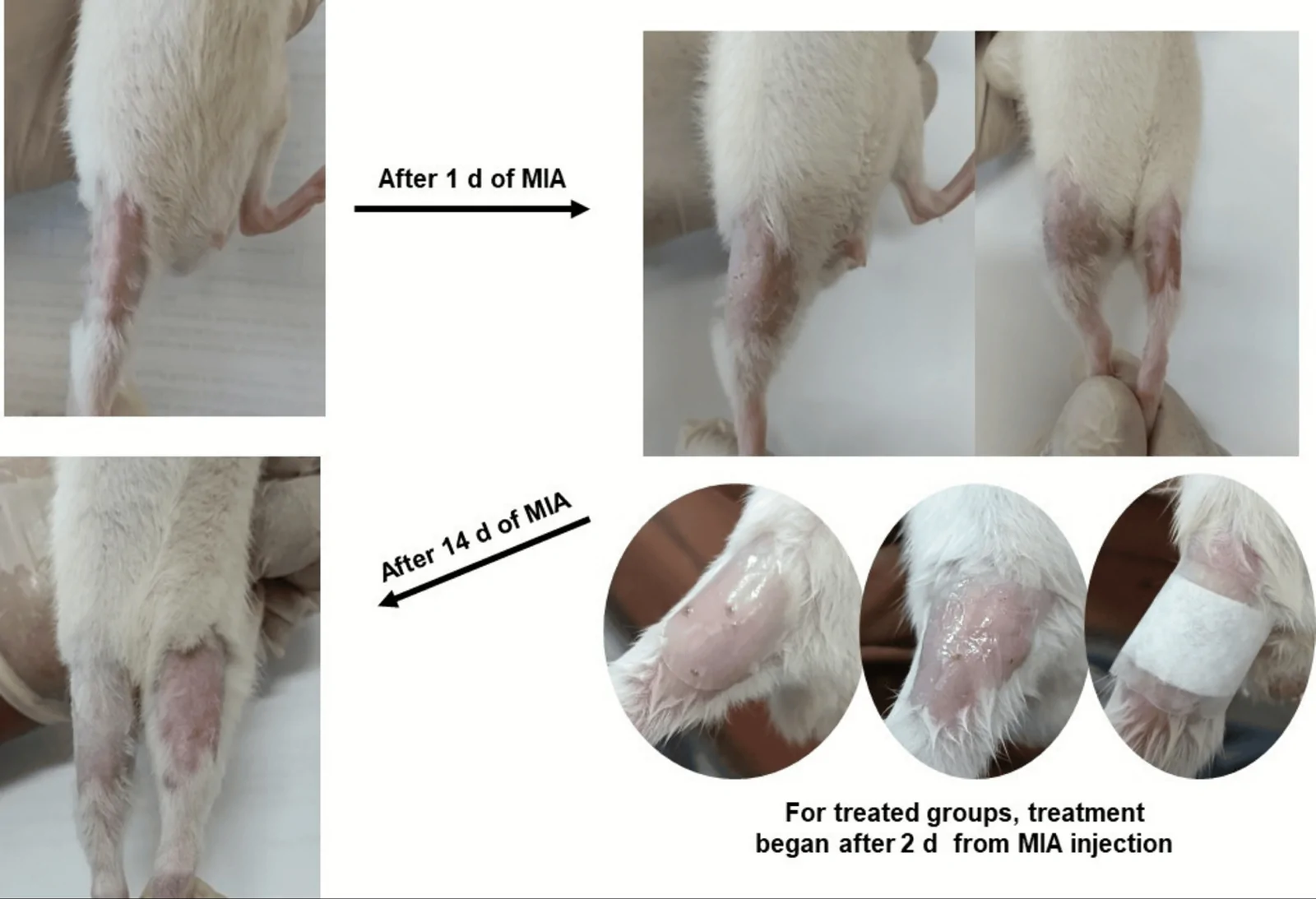
Gross images of rat right knees (n = 5) demonstrating MIA-induced osteoarthritic swelling and its alleviation after 14-day treatment with ETX-NCs transdermal patches

**Fig. 7:**
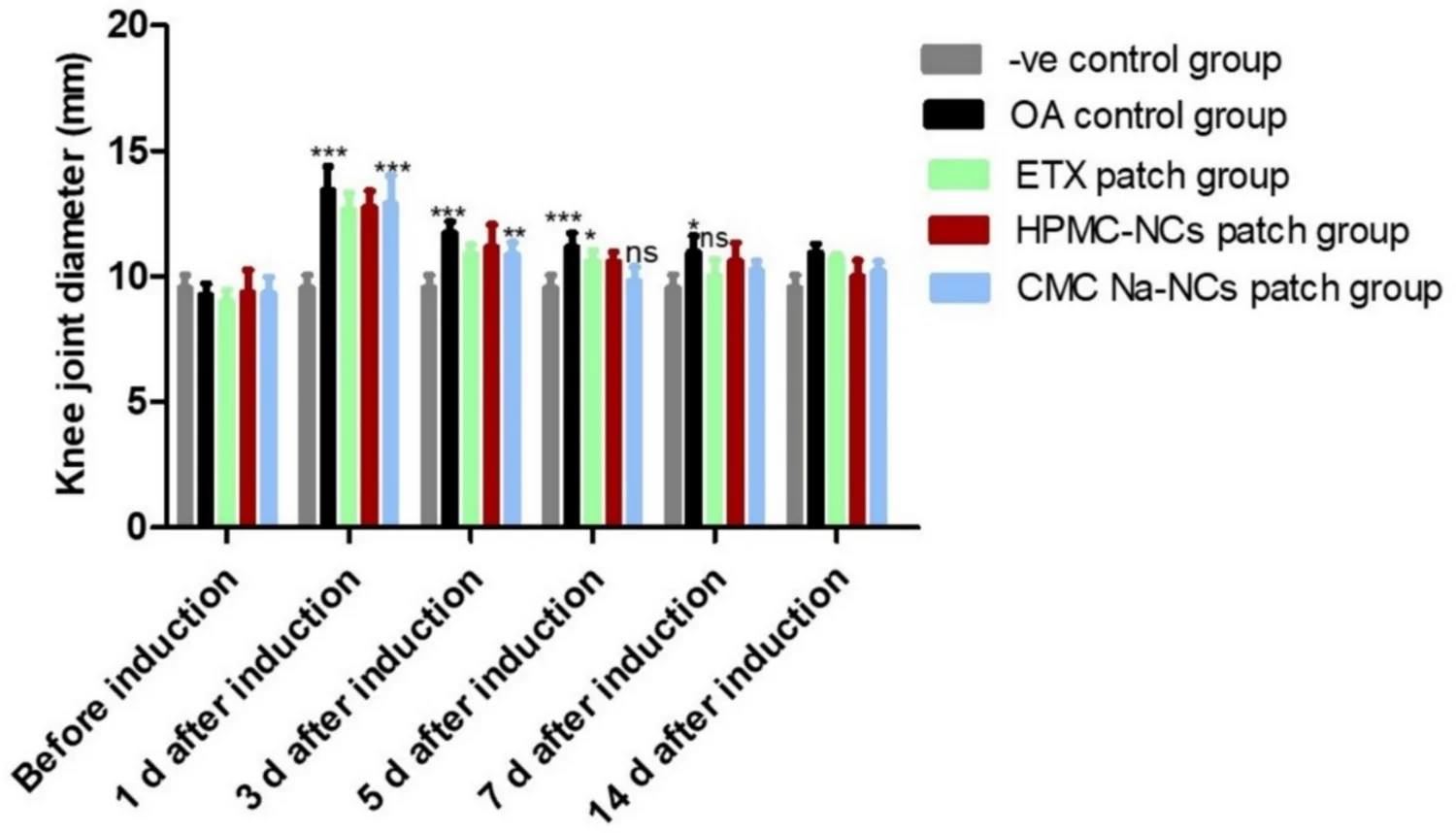
Effect of ETX-NCs patches on joint swelling caused by MIA induced OA, All data are reported as mean ± SD (n = 5). One way ANOVA followed by the Newman–Keuls post hoc test was used for statistical analysis;* (p < 0.05), ** (p < 0.01), *** (p < 0.001), ns (non-significant) compared to -ve control group

Joint diameter was measured as an objective indicator of inflammation severity and therapeutic response following MIA induced osteoarthritis. As shown in Figs. 6 and 7, significant increase in joint diameter was observed 24 h after intra-articular MIA injection in all injected groups compared with the saline-injected negative control (p < 0.001). Treatments were initiated on day 2 post-injection and administered once daily for 14 days. By day 5, rats treated with the CMC Na–NCs patch exhibited a marked reduction in joint diameter to levels comparable with the negative control, without significant difference. Similarly, the HPMC–NCs and ETX patch treated groups showed significant reductions in joint swelling relative to the OA control (p < 0.05), whereas the untreated OA group continued to display pronounced swelling (p < 0.001) (Fig. 7). By day 7, joint diameters in all treated groups returned to near-normal values, with no significant differences from the negative control, while the OA control group still exhibited residual but significant swelling (p < 0.05). These results revealed improvement compared to findings of Wen et al., who observed decreased joint swelling, at 12–21 weeks after surgery, in surgically induced OA rats treated orally with ETX three times weekly from weeks 8 to 21 post-surgery [10].

#### Serum analysis

**Fig. 8:**
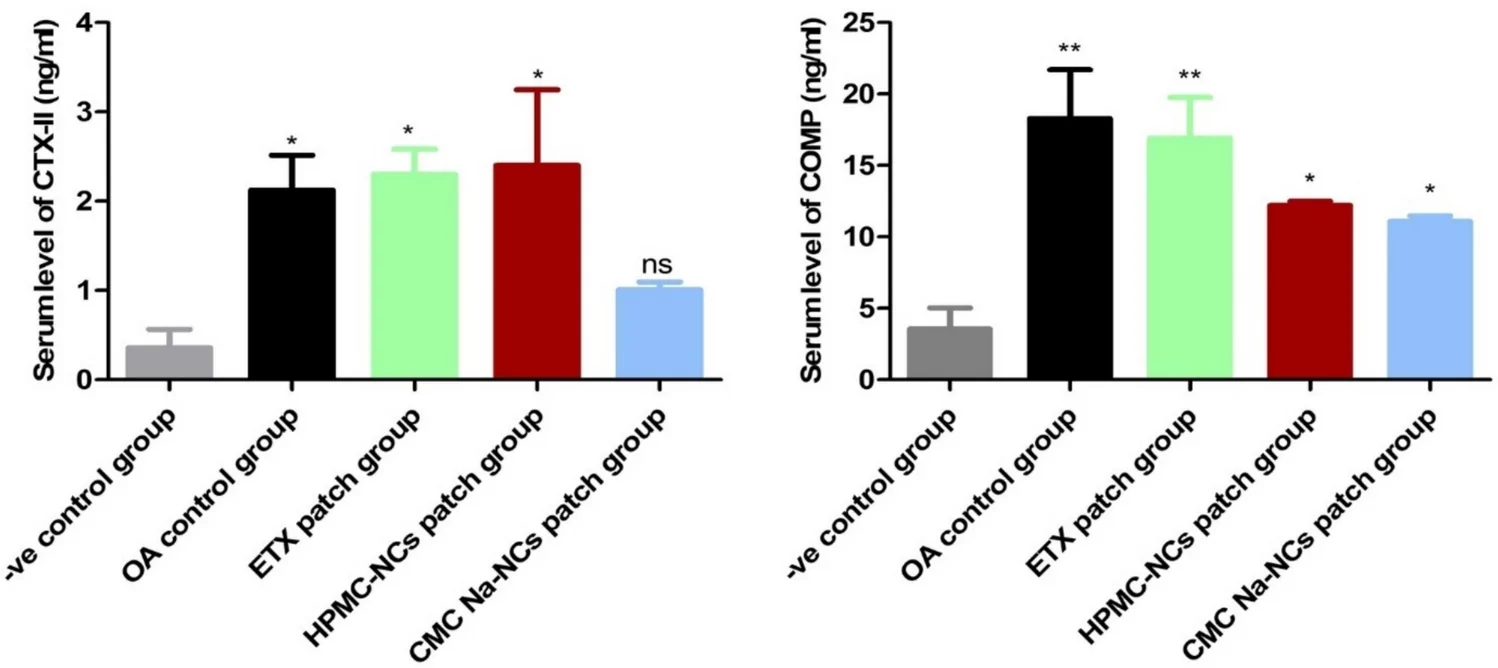
Serum levels of CTX-II and COMP for all groups, values are expressed as the mean ± SD (n = 5). One way ANOVA followed by the Newman–Keuls post hoc test was used for statistical analysis;* (p < 0.05), ** (p < 0.01), ns (non-significant) relative to the negative control group

Serum analysis of cartilage oligomeric matrix protein (COMP) and the C-telopeptide of type II collagen (CTX-II) was performed to quantitatively evaluate cartilage breakdown and osteophyte-related joint degeneration during osteoarthritis (OA) progression. As shown in Fig. 8, the untreated OA control group exhibited significantly elevated serum levels of COMP (p < 0.01) and CTX-II (p < 0.05) compared with the negative control group. In contrast, treatment with ETX-NCs patches significantly reduced the levels of both biomarkers. Collectively, these results highlight the therapeutic potential of ETX-NCs patches in attenuating OA-associated cartilage degradation.

#### Radiographic assessment

**Fig. 9:**
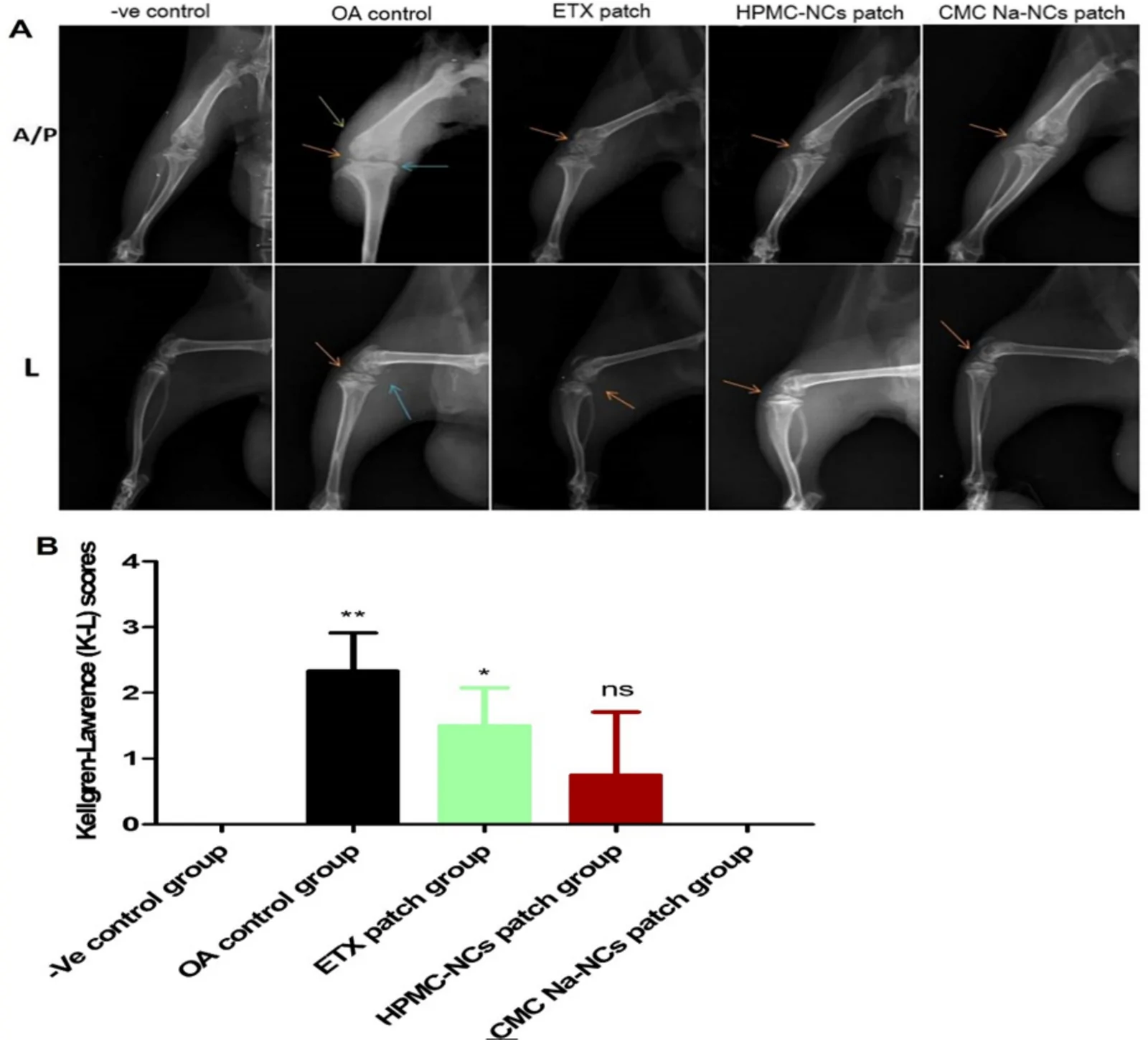
(**A**) Radiographic images of all examined groups (n = 5) in anteroposterior (A/P) and lateral (L) views, where -ve control group showed normal radiographs of smooth articular surfaces with normal joint space. However, the images of OA control group showed increased joint opacity (Green arrow), osteophyte formation, rough surfaces of the femoral condyles and proximal tibia (orange arrow) and narrowing of the joint space (blue arrow). On the other hand, the X-ray images of the ETX patch treated group showed almost normal joint space with little lysis of the femoral articular surface and few osteophytes (orange arrow). Regarding HPMC-NCs patch group, the joint capsule space showed almost normal, the femoral condyle and proximal tibia were nearly smooth surface (orange arrow). In the CMC Na-NCs patch treated group, articular surfaces appeared smooth without any bone irregularities or erosions nor narrowing of the joint space (orange arrow). (**B**) represents the Kellgren-Lawrence (K-L) scores of each group compared to -ve control group. one way ANOVA followed by the Newman–Keuls post hoc test was used for statistical analysis;* (p < 0.05), ** (p < 0.01), ns (non-significant), Results are expressed as mean values ± SD

Radiographic evaluation was performed to assess structural changes in the knee joint and to monitor the extent of osteoarthritic degeneration and treatment-related protection. On day 14, the negative control group (Fig. 9A) maintained normal knee joint anatomy, with preserved joint space, smooth articular surfaces, and no evidence of osteophyte formation, subchondral sclerosis, or surface irregularities, indicating healthy joint architecture. In contrast, the untreated OA control group exhibited characteristic osteoarthritic changes, including marked joint space narrowing, marginal osteophyte formation, increased intra-articular radiopacity suggestive of joint effusion, and rough, irregular articular surfaces consistent with subchondral bone degradation. Treatment with ETX patch partially ameliorated these changes, as evidenced by largely preserved joint space, mild articular surface irregularities, minimal subchondral lysis, and the presence of few small marginal osteophytes. Similarly, the HPMC-NCs patch treated group demonstrated near-normal joint radiodensity and surface regularity, with only minor capsular distension and limited marginal osteophytic changes in some animals. Notably, the CMC-Na-NCs patch treated group showed the greatest protective effect, with preserved joint space, smooth and intact articular surfaces, absence of joint effusion or periarticular swelling, and no detectable osteophyte formation, indicating effective preservation of joint structural integrity. These observations were supported by Kellgren–Lawrence (K–L) scoring (Fig. 9B), which showed significant osteoarthritic changes in the OA control group (p < 0.01). The ETX patch treated group exhibited only mild radiographic alterations (p < 0.05) compared with the negative control. In contrast, the HPMC-NCs and CMC Na-NCs treated groups showed no significant differences from healthy control.

#### Histopathology

**Fig. 10:**
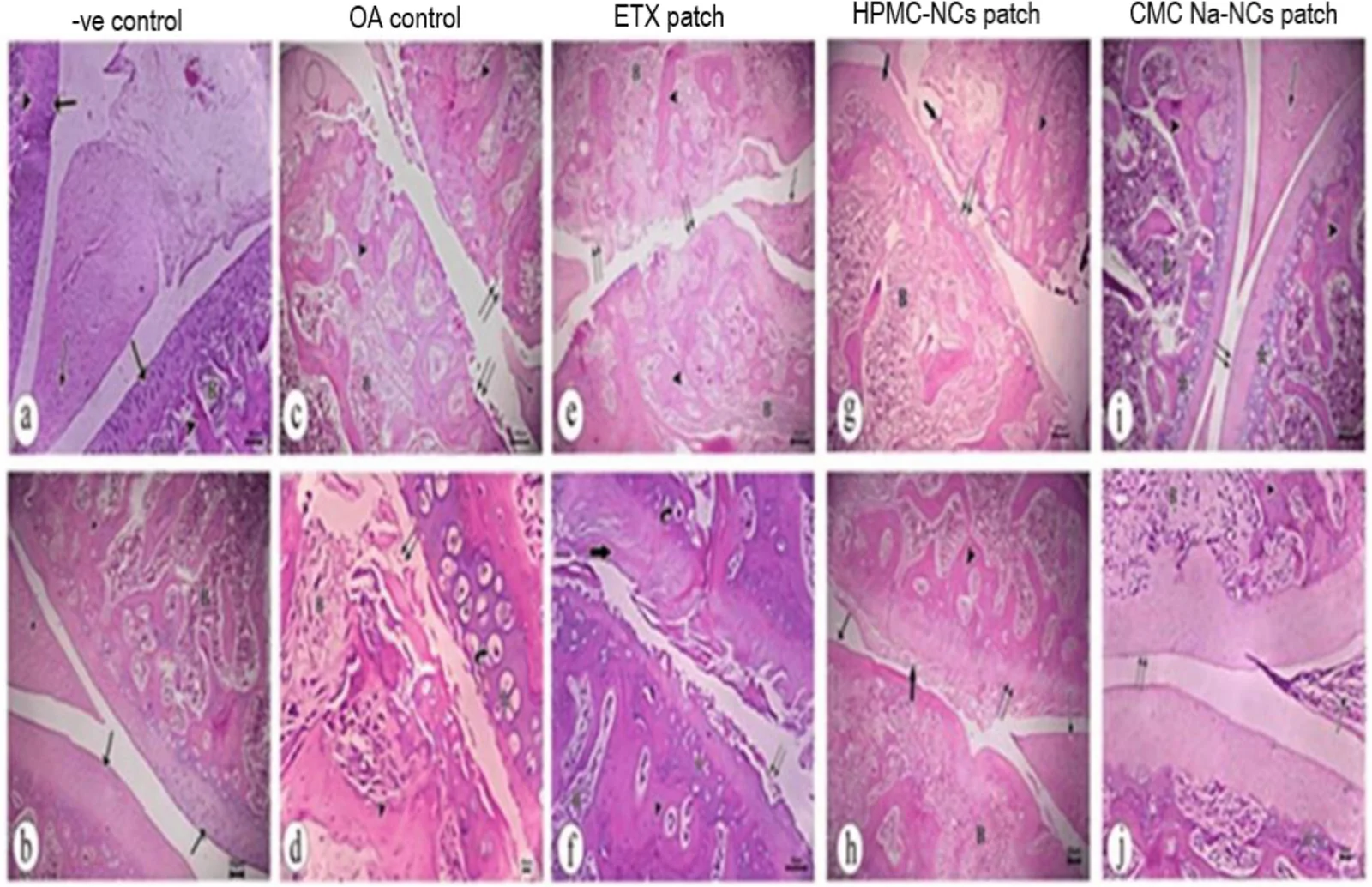
A photomicrograph of knee joints (n = 5): sections (**a**,**b**) from –ve control group showing normal cytoarchitecture of articular cartilage with chondrocytes inside lacunae (arrow), synovial membrane (wavy arrow), subchondral bone (arrowhead) and bone marrow spaces (B). Sections from OA control group (**c**,**d**) show denuded articular surface with deep fissures (double arrow), empty chondrocytes (astrix), chondrocytes with hyperchromatic nuclei (curved arrow), degenerated synovial membrane cells (circle), disorganized subchondral bone trabeculae (arrowhead) and widen bone marrow spaces (B). Sections from ETX patch (**e**,**f**) show affection of joints like OA control changes with the appearance of fibrosis of the articular cartilage (thick arrow). Sections from HPMC-NCs patch group (**g**,**h**) show apparent preservation of the articular cartilage (double arrow), decreased fibrosis (thick arrow), apparent normally organized subchondral bone(arrowhead) and bone marrow spaces (B). Sections from CMC Na-NCs patch (**i**,**j**) show apparent normal histological structure of the knee joint, preserved articular cartilage (double arrow), synovial membrane, apparent normally organized subchondral bone (arrowhead) and bone marrow spaces (B)

Histopathological evaluation was conducted to assess cartilage integrity, subchondral bone architecture, and synovial membrane alterations associated with osteoarthritis progression and treatment effects. As shown in Fig. 10, knee joint sections from the negative control group exhibited smooth articular surfaces, intact hyaline cartilage with normal chondrocytes in lacunae, preserved subchondral bone architecture, and a normally organized synovial membrane (Fig. 10 a,b). In contrast, osteoarthritic (OA control) group revealed loss of surface smoothness with evident cracks and fissures in the articular cartilage. The cartilage was markedly destructed and degenerated chondrocytes with hyperchromatic, pyknotic nuclei were observed. The subchondral bone exhibited structural disorganization. The synovial membrane appeared disrupted with loss of synoviocytes. Moreover, bone marrow spaces were noticeably widened (Fig. 10, c, d). The ETX patch treated group showed histopathological features largely similar to the OA control group, with additional evidence of cartilage fibrosis (Fig. 10 e,f). HPMC-NCs patch treated group exhibited partial cartilage preservation with reduced fibrosis and relatively organized subchondral bone and bone marrow spaces, although degenerative changes persisted in some areas (Fig. 10, g,h). Notably, the CMC Na-NCs patch treated group displayed near-normal joint histology, characterized by smooth articular surfaces, preserved chondrocytes, restored subchondral bone architecture, and an intact synovial membrane, indicating effective protection against OA-related structural damage. The OARSI scoring system corroborated these findings, as ETX-NCs patches did not produce scores significantly different from the negative control group, while OA control and ETX patch groups showed significantly elevated scores (p < 0.01 and p < 0.05, respectively), Table 7. Comparable findings were reported by Arunkumar et al., who developed an intra-articular chitosan microparticles-based etoricoxib thermogel; histopathological evaluation demonstrated marked improvement in joint by the end of the third week of treatment [80].

**Table 7:** OARSI-based histological scoring of knee joints in rats with MIA-induced osteoarthritis

Group	Grade	Stage	Score
-ve control group	0.00	0.00	0.00
OA control	5.60 ± 0.54	3.80 ± 0.44	21.20 ± 2.68^**^
ETX patch	5.25 ± 0.95	3.75 ± 0.50	19.50 ± 3.41^*^
HPMC-NCs patch	3.25 ± 0.50	2.75 ± 0.50	9.00 ± 2.44
CMC Na-NCs patch	0.40 ± 0.54	0.40 ± 0.54	0.40 ± 0.54

Data are presented as mean ±SD (n=5). Kruskal-Wallis was used for statistical analysis followed by Dunn’s multiple comparisons, ^*^ indicates p<0.05, and ^**^ indicates p<0.01 relative to -ve control group

As observed in our study, the histopathological findings were consistent with radiographic assessments and serum markers of cartilage degradation (COMP and CTX-II). In the untreated OA control group, cartilage deterioration may resulted from MIA-induced inhibition of chondrocyte glycolysis, leading to energy depletion and apoptosis [81]. The loss of viable chondrocytes resulted in upregulation of catabolic genes and matrix-degrading enzymes such as matrix metalloproteinases (MMPs). This triggered inflammatory mediators such as prostaglandin E₂ (PGE₂) and activated nuclear factor-κB (NF-κB) signaling, promoting pain, chronic inflammation, and further cartilage matrix degradation [82–84]. In contrast, treatment with ETX patch, HPMC-NCs patch, or CMC Na-NCs patch reduced cartilage damage through COX-2 inhibition, suppressing cartilage-degrading pathways and alleviating OA symptoms, consistent with previous reports by He et al., Sun et al., and Timur et al., [85–87]. Notably, the nanocrystal formulations (HPMC-NCs and CMC Na-NCs patches) provided superior protection compared to conventional ETX patches, reflecting enhanced permeation, bioavailability, and stronger anti-inflammatory effects at a lower dose (30 mg/day) over a shorter 14-day treatment period.

## Conclusion

Formulation of etoricoxib (ETX) nanocrystals into various transdermal patch matrices (HPMC, CMC Na, PVA, and sodium alginate) followed by pharmaceutical optimization led to the development of highly effective delivery systems. Among these, the HPMC-NCs and CMC Na-NCs patches exhibited the most favorable drug release and skin permeation profiles. These optimized formulations significantly enhanced the therapeutic efficacy of ETX in alleviating osteoarthritic symptoms and in mitigating the progression and exacerbation of the disease. The pharmacological findings were supported by serum analyses of cartilage degradation biomarkers (COMP and CTX-II), radiographic assessments, and histopathological examinations. Moreover, the results demonstrated superior performance of the nanocrystal ETX formulations compared to the conventional (non-nanocrystal) form, confirming the advantage of the nanocrystal-based transdermal delivery approach.

## Data Availability

The datasets analyzed during the current study are available from the corresponding author on reasonable request.
